# Supplementary material for: Platelet Endothelial Cell Adhesion Molecule 1 (CD31) Is Essential for Clostridium perfringens Beta-Toxin Mediated Cytotoxicity in Human Endothelial and Monocytic Cells
Source: Toxins (Basel). 2021 Dec 13;13(12):893. doi: 10.3390/toxins13120893 (PMC8703487; doi:10.3390/toxins13120893)
Supplement: Supplementary file 1 [file toxins-13-00893-s001.zip › toxins-1493407 -su-send for xml-12.20.pdf]

Supplementary Materials: Platelet Endothelial Cell Adhesion Molecule 1 (CD31) Is Essential for *Clostridium Perfringens* Beta-Toxin Mediated Cytotoxicity in Human Endothelial and Monocytic Cells.

Basma Tarek, Julia Bruggisser, Filippo Cattalani, and Horst Posthaus

Table S1. Amino acid sequence of recombinant N-terminally His-Tagged CPB.

**His_6_ CPB**
**HHHHHH**NDIGKTTTITRNKTSDGYTIITQNDKQIISYQSVDSSSKNEDGFTASIDARFIDDKYSSE MTTLINLTGFMSSKKEDVIKKYNLHDVTNSTAINFPVRYSISILNESINENVKIVDSIPKNTISQKT VSNTMGYKIGGSIEIEENKPKASIESEYAESSTIEYVQPDFSTIQTDHSTSKASWDTKFTETTRGNY NLKSNNPVYGNEMFMYGRYTNVPATENIIPDYQMSKLITGGLNPNMSVVLTAPNGTEESIIKV KMERERNCYYLNWNGANWVGQVYSRLAFDTPNVDSHIFTFKINWLTHKVTAI

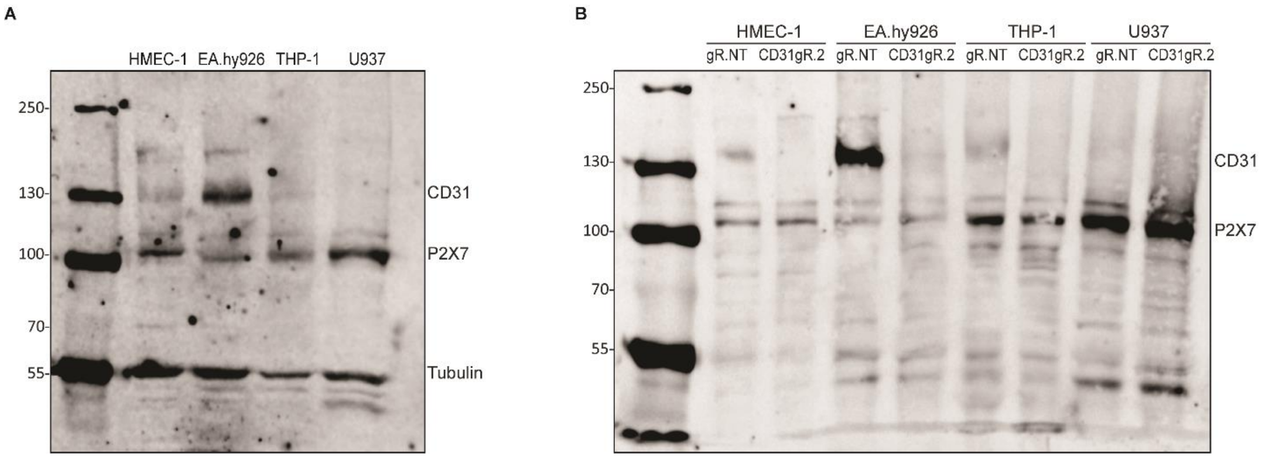

Figure S1. P2X7 expression in CD31 proficient and deficient cells. (**A**) Western blots of HMEC-1, EA.hy926, THP-1, and U937 probed first with CD31 and Tubulin antibodies, thoroughly washed with TBST, followed by incubation with P2X7 antibodies. (**B**) Western blots of HMEC-1, EA.hy926, THP-1, and U937 ΔCD31gR.2 polyclonal cell populations incubated first with CD31 antibodies, blots were thoroughly washed with TBST, followed by incubation with P2X7 antibodies. As a control, cells transformed with non-target gRNA (gR.NT) were used.

523 - 528 536 - 546

homo CD31 **Ig6** domain APVDEVQISILSSKVVESGEDIVLQCAVNEGSGPITYKFYREKEGKPFY

homo CD31 **Ig6** domain QMTSNATQAFWTKQKASKEQEGEYYCTAFNRANHASSVPRSKILT

Figure S2. Amino acid sequence of the human CD31 Ig6 domain. The epitope recognized by mAb 4G6 is labelled in red. In Blue, the conserved epitope among mouse, human and pig, previously described by Bruggisser *et. al* 2020 to be essential for interaction with CPB. Amino acid numbering refers to full length CD31 with signal peptide included.
